# Pneumococcal Vaccine Uptake in Adults Before and After Hospitalization for Pneumococcal Infections in Hong Kong, 2015 to 2024

**DOI:** 10.3390/vaccines13050541

**Published:** 2025-05-19

**Authors:** King-Pui Florence Chan, Ting-Fung Ma, James Chung-Man Ho, Ivan Fan-Ngai Hung, Mary Sau-Man Ip, Pak-Leung Ho

**Affiliations:** 1 Department of Medicine, Queen Mary Hospital, University of Hong Kong, Hong Kong SAR, China; ckp663@ha.org.hk (K.-P.F.C.); jhocm@hku.hk (J.C.-M.H.); ivanhung@hku.hk (I.F.-N.H.); msmip@hku.hk (M.S.-M.I.); 2 Department of Statistics, University of South Carolina, Columbia, SC 29201, USA; tingfung@mailbox.sc.edu; 3 Department of Microbiology, University of Hong Kong, Hong Kong SAR, China; 4 Carol Yu Centre for Infection, University of Hong Kong, Hong Kong SAR, China

**Keywords:** pneumococcal vaccines, pneumococcal disease, vaccine uptake, hospitalization

## Abstract

**Background/Objectives**: Vaccination is a key preventive measure against pneumococcal disease, but uptake rates remain low in high-risk populations. Limited information exists on pneumococcal vaccine uptake in individuals with a history of pneumococcal disease. This study aims to assess pneumococcal vaccine uptake and the factors associated with it in patients hospitalized for pneumococcal disease, before and after hospitalization, across time periods before, during, and after the COVID-19 pandemic. **Methods**: Data for patients aged ≥18 years who were hospitalized for pneumococcal disease between 2015 and 2024 were extracted from the Hospital Authority’s territory-wide electronic medical record database. The uptake of pneumococcal vaccines in subgroups aged 18–64 years and ≥65 years, with and without risk conditions, both before and after hospitalization for pneumococcal disease, was assessed, followed by multivariate analyses of the factors associated with vaccination uptake by logistic regression models. **Results**: This study included 5517 patients hospitalized for pneumococcal disease. Prior to hospitalization, the vaccination uptake among the eligible patients was 20.5%, with only 8.1% fully vaccinated, despite the majority (87.9%) having previous hospitalizations (subgroup medians 3–9 times) or outpatient clinic visits (subgroup median 61–107 times). After discharge, during a median follow-up of 1.85 years, almost all the eligible patients (98.4%) received subsequent inpatient (subgroup medians 3–4 times) and outpatient (subgroup medians 21–28 times) care, but only 32.2% of the eligible patients received the vaccine. Factors associated with increased vaccine uptake post-discharge included age ≥75 years (OR 1.6), ≥10 subsequent hospitalizations (OR 2.1), and ≥10 subsequent clinic visits (OR 55.9). Vaccination rates within 12 months post-discharge were significantly lower in the patients hospitalized during the COVID-19 pandemic (3.5%) compared to the baseline (11.6%) and post-COVID-19 (6.6%) periods. **Conclusions**: The uptake of the pneumococcal vaccine before hospitalization for pneumococcal disease was low and continued to be suboptimal post-discharge. Numerous vaccination opportunities were missed in both the inpatient and outpatient settings. These findings indicate a need to improve vaccination strategies.

## 1. Introduction

Pneumococcal disease, including pneumococcal pneumonia and invasive pneumococcal disease (IPD), is a major cause of morbidity and mortality on a global scale, including in Hong Kong [1,2]. Vaccination plays a crucial role in preventing pneumococcal disease [3,4,5]. Currently, both types of available pneumococcal vaccines rely on inducing serotype-specific antibodies against the capsule [4]. In adults, both the 23-valent polysaccharide pneumococcal vaccine (23vPPV) and the pneumococcal conjugate vaccine (PCV) demonstrate efficacy and effectiveness against vaccine-type invasive pneumococcal disease and pneumococcal pneumonia [4,5]. Among these vaccines, 23vPPV elicits T-independent immune responses that are short-lived without memory B cell induction [6], whereas PCV triggers a T-dependent immune response with immune memory [3,7]. It is recognized that the first dose of PCV13 primes an improved response to a subsequent dose of 23vPPV, while the initial 23vPPV dose diminishes the effect of subsequent PCV13 dosing [8]. Thus, national recommendations suggest administering PCV13 (or a higher valent PCV) first when both PCV and 23vPPV are recommended [9,10]. Currently, 23vPPV and various formulations of PCV (PCV13, PCV15, PCV20, and PCV21) are approved for adult use [10,11]. The newer PCVs cover more serotypes and have the potential to prevent more pneumococcal disease, prompting countries to reassess their recommendations on pneumococcal vaccination [12,13,14].

In Hong Kong, pneumococcal vaccination has been included in the adult immunization program since 2014 [15]. This followed the introduction of the PCV in the routine infant immunization program in 2009 [16,17]. Since August 2024, either 23vPPV alone or PCV15 in series with 23vPPV is recommended for adults aged ≥65 years (age-based) and for adults aged 18–64 years with conditions that increase the risk for pneumococcal disease (risk-based) [15]. This replaced a previous recommendation of 23vPPV with or without PCV13 for these groups [18]. Under the program, eligible individuals receive free vaccination in the public sector or a subsidy for vaccination in the private sector. Those who are not eligible may choose to receive any registered pneumococcal vaccines (23vPPV, PCV15, PCV20) for protection against pneumococcal disease [18].

Although pneumococcal vaccination offers significant benefits, the uptake of the vaccine remains suboptimal. Limited data exist on pneumococcal vaccination rates among individuals with prior pneumococcal infection, particularly those with indications for vaccination [19,20]. In the United States, a study examining adults aged 18 years and above with IPD between 2001 and 2003 found that 52% of cases with a vaccine indication were not vaccinated, where nearly all unvaccinated IPD patients with a vaccine indication had one or more missed opportunities for vaccination documented [19]. In another U.S. study involving 229 cases of IPD from 2014 to 2019, only 14% of patients were appropriately vaccinated. Additionally, more than half of the patients who developed IPD had missed vaccination opportunities either during their previous hospitalization or at an outpatient visit [20]. However, these studies did not explore the uptake of pneumococcal vaccine as secondary prevention following the patients’ hospitalization for pneumococcal disease. In patients with IPD, the risk of recurrent attacks outweighed that of primary IPD in the general population [21,22]. A study conducted on IPD cases in Australia from 1991 to 2016 revealed that the incidence of recurrent IPD was 27 times higher than the population rate of primary IPD during this timeframe [21]. Given the high risk of recurrence, a history of IPD is considered an indication for pneumococcal vaccination. Although Streptococcus pneumoniae is recognized as one of the leading causes of recurrent pneumonia [23], a history of pneumococcal pneumonia, excluding IPD, is not considered an indication for pneumococcal vaccination in most national programs [9,13].

In Hong Kong, the annual number of cases of pneumococcal disease requiring hospitalization in the years 2015 to 2004 ranged from 271 to 735 cases per year, with an incidence rate of 4.20 to 11.41 per 100,000 person-years. The annual number (572 cases) and incidence rate (8.7, [95% CI 8.0–9.4] cases per 100,000 person-years) in 2023 was higher than those in 2020–2022 (271–392 cases and 4.2–6.0 cases per 100,000 person-years) but remained lower than baseline average for years 2015–2019 (699 cases and 11.0 [95% CI 10.6–11.3] cases per 100,000 person-years) [24,25]. The annual incidence was higher in those aged ≥75 years, ranging from 32.1 to 56.3 per 100,000 person-years, compared to 0.3 to 2.9 per 100,000 person-years in those aged 18–49 years. Mortalities during inpatient stay were 88 to 146 (16.1–35.6%) annually. Mortality was higher in those aged ≥75 years, ranging from 20.6–39.4%, compared to 1.3–19.0% in those aged 18–49 years [24,25].

During the Coronavirus disease (COVID-19) pandemic, a temporary decline in pneumococcal disease incidence was noted, followed by a rebound in cases [2]. In the United Kingdom, there was an increase in IPD incidence post-pandemic, with a rise in serotypes contained in the PCV13 compared to the pre-COVID-19 period, but it remained below the pre-PCV13 levels [26]. Global online interest in pneumococcal vaccines surged during the COVID-19 pandemic [27]. However, vaccination rates varied globally during this period, with reports of increased vaccination rates in some regions, such as Portugal, and lower rates in others, such as Argentina and Canada [28,29,30]. These observations prompted us to use electronic inpatient health records in Hong Kong to study pneumococcal vaccine uptake in a cohort of patients with pneumococcal infections from 2015 to 2024 [24]. The pneumococcal vaccine uptake was assessed before and after hospitalization for pneumococcal infections across time periods before, during, and after the COVID-19 pandemic.

## 2. Materials and Methods

### 2.1. Study Design

This is a follow-up study on pneumococcal vaccination status based on a previously published cohort of patients who were hospitalized because of pneumococcal infection [24]. The pneumococcal vaccination status before and after hospitalization for pneumococcal disease was collected and analyzed alongside demographics, number of hospitalizations, and clinic attendance before and after hospitalization for pneumococcal disease. The study period was divided into three phases: baseline (January 2015 to December 2019), COVID-19 pandemic (January 2020 to December 2022), and post-pandemic (January 2023 to August 2024).

Data were extracted from the Hospital Authority’s territory-wide electronic medical record database, Clinical Data Analysis and Reporting System (CDARS), where patient identities were anonymized [31,32]. The Hospital Authority is a statutory body that manages public hospital services, providing 90% of inpatient services in Hong Kong, with 8 million specialist outpatient clinical attendances and 9 million primary care attendances yearly [31,33].

In Hong Kong, the Scientific Committee on Vaccine Preventable Diseases regularly issues recommendations regarding the use of pneumococcal vaccines [18]. Adults aged 18–64 years and ≥65 years with high-risk conditions are recommended to receive two doses of pneumococcal vaccines [18]. Currently, this consists of one dose of PCV15, followed by one dose of 23vPPV one year later. For those aged ≥65 years without high-risk conditions, one dose of 23vPPV is recommended. Adults aged 19 to 64 years without high-risk conditions are not eligible for pneumococcal vaccination. Patients were considered to be eligible when there was an age-based or risk-based vaccine indication. High-risk conditions that qualified for pneumococcal vaccination, including history of invasive pneumococcal disease, chronic cardiovascular diseases (including myocardial infarction, heart failure), chronic lung disease (including chronic obstructive pulmonary disease), chronic liver disease (including liver cirrhosis and liver failure), chronic kidney disease, diabetes mellitus, immunocompromised states (including malignancy, human immunodeficiency virus infection), and chronic neurological conditions (including hemiplegia and previous stroke), were collected for analysis. During the study period, only PCV13, PCV15, and 23vPPV were available. To assess pneumococcal vaccination prior to developing pneumococcal infection, the time period for immunization documentation was extended from January 2005 to August 2024 to cover a minimum 10-year period prior to development.

The pneumococcal vaccination status was assessed using immunization records from January 2005 to November 2024 as follows: fully vaccinated (2 doses of any available vaccine for those aged 18–64 years and ≥65 years with high-risk conditions, or 1 dose of any available vaccine for those aged ≥65 years without high-risk conditions), partially vaccinated (1 dose for those aged 18–64 years and ≥65 years with high-risk conditions), and unvaccinated (never vaccinated) [15].

The study period was divided into three phases: baseline (January 2015 to December 2019), COVID-19 pandemic (January 2020 to December 2022), and post-pandemic (January 2023 to August 2024). Outpatient clinic attendances and hospitalizations were tracked before and after the initial hospitalization for pneumococcal disease, with the censor date set to 30 November 2024. The number of clinic visits and hospitalizations was grouped into categories, i.e., 1–5 times, 6–10 times, and more than 10 times, to assess the correlation between attendance frequency and pneumococcal vaccination status. A vaccination opportunity was identified as a visit to a clinic (primary care or specialist) or a hospital admission between January 2011 and August 2024. If a patient was eligible for vaccination but did not receive one during the visit or admission, it was regarded as a missed opportunity.

### 2.2. Outcomes

The primary outcome of this study was to assess the uptake of the pneumococcal vaccine both pre- and post-hospitalization for pneumococcal disease. The secondary outcomes included factors associated with pneumococcal vaccination before hospitalization for pneumococcal disease, during follow-up post-discharge, and the percentage of patients receiving the pneumococcal vaccine after discharge during the baseline, COVID-19, and post-COVID-19 periods.

### 2.3. Statistical Analysis

The uptake of the pneumococcal vaccine, both pre- and post-hospitalization for pneumococcal disease, was treated as a binary outcome (vaccinated and unvaccinated) and assessed by inference on odds ratios and contingency tables. The analysis was based on the whole population followed by subgroup analysis based on age (18–64 years vs. ≥65 years) and with or without risk conditions, classified into 18–64 years without risk conditions, 18–64 years with risk conditions, ≥65 years without risk conditions and ≥65 years with risk conditions. The data were compared between time periods (baseline, COVID-19 pandemic, and post-pandemic).

Multivariate analyses were conducted using logistic regression models for inference on adjusted odds ratios (ORs). Other key patient key features, namely, age groups (<65 y, 65–74 y, ≥75 y), gender, number of risk conditions, hospitalization before and after pneumococcal disease (1–5 times, 6–10 times, and ≥10 times), and clinic attendance before and after pneumococcal disease (1–5 times, 6–10 times, and ≥10 times), were treated as model covariates to adjust for their impact on the outcome [34].

Confidence intervals of the regression coefficients were estimated using their large sample distributions. Based on the relationship between adjusted ORs and regression coefficients, the adjusted ORs of the key factors, including time period, were estimated. The corresponding confidence intervals were estimated based on the Delta method [35]. The *p*-values were used to assess the statistical significance of the adjusted ORs under the null hypothesis. Bayesian logistic regression was implemented when necessary as a robust alternative to the simple pseudo-count method [36]. This approach incorporated prior information and controlled the covariate effects simultaneously. Independent Cauchy distributions were chosen as the prior distributions of the regression coefficients for the Bayesian logistic regression for statistical inference using the posterior distributions.

## 3. Results

**Figure 1:**
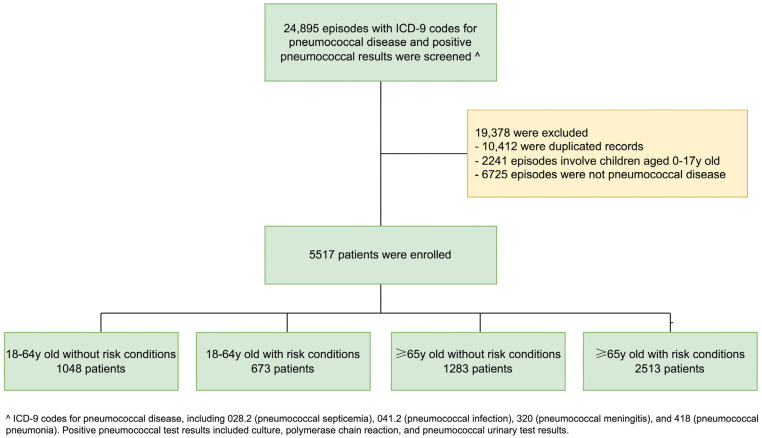
Patient selection flowchart.

Based on a previously published cohort of patients with pneumococcal infection who required hospitalization, 5517 patients were enrolled, which included 1084 (19.6%) patients aged 18–64 years without risk conditions, 673 (12.2%) patients aged 18–64 years with risk conditions, 1283 patients (23.3%) aged ≥65 years without any risk conditions, and 2513 (45.6%) patients aged ≥65 years with risk conditions (Figure 1) [24].

### 3.1. Uptake of Pneumococcal Vaccination Among the Study Population

**Figure 2:**
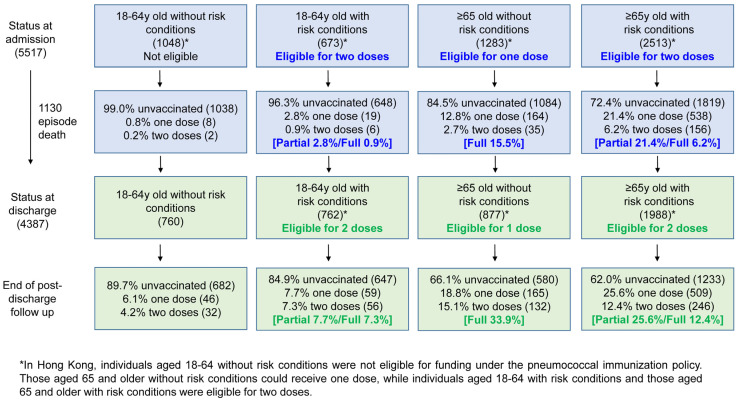
Pneumococcal vaccination status among patients hospitalized for pneumococcal infection and during the post-discharge follow-up. The number of patients is indicated inside round brackets, while the percentages of partial and full vaccination are given inside square brackets. The number of patients in the four groups at discharge was adjusted for episode death and a history of invasive pneumococcal disease as a risk condition.

Upon hospitalization for pneumococcal infection, 4469 (81%) of the 5517 patients were deemed eligible for pneumococcal vaccination (Figure 2). The remaining 1048 (19%) patients without risk conditions, including 458 (8.3%) patients aged 18–49 years and 590 (10.7%) patients aged 50–64 years, were not eligible. The uptake rate of any dose of a pneumococcal vaccine before developing pneumococcal disease was 16.8% (928/5517) in all the patients and 20.5% (918/4469) among the eligible patients. Among the eligible patients, only 8.1% (361/4469) were deemed fully vaccinated, ranging from 0.9% in those aged 18–64 years with risk conditions to 15.5% in those aged ≥65 years without risk conditions and 6.2% in those aged ≥65 years with risk conditions (Figure 2). There were variations in the vaccine uptake rate based on the patients’ admission year during the study period. The partial and full vaccination rates ranged from 10.1% to 15.2% and 3.5% to 11.9%, respectively, while 73.6% to 84.7% of the patients were unvaccinated (Figure S1).

There were 1130 deaths among the patients hospitalized for pneumococcal infection. Among the 4387 survivors, 579 patients (223 patients aged 18 to 64 years old and 356 patients from the≥65-year-old group) had a new indication for vaccination or an additional dose due to a history of invasive pneumococcal disease. Considering this, the percentage of discharge patients eligible for vaccination or additional doses was 76.5% (3356/4387).

**Figure 3:**
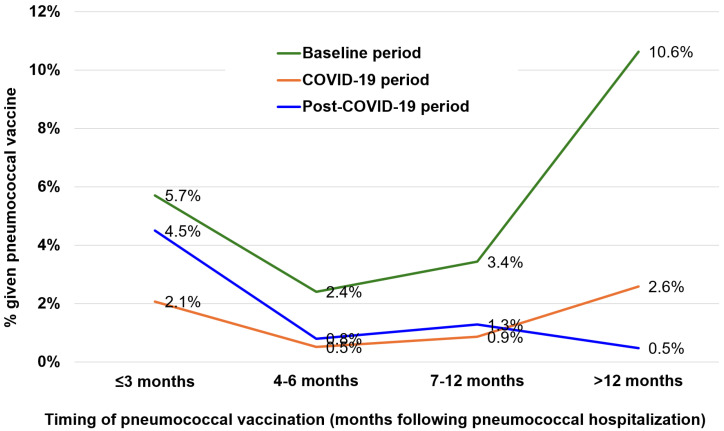
Proportion of eligible patients given a pneumococcal vaccine after discharge. The timing of pneumococcal vaccination is shown as month intervals after discharge for patients hospitalized during the baseline (2015–2019), COVID-19 (2020–2022), and post-COVID-19 periods (2023–2024). *p* < 0.001 for all comparisons for the ≤3 month, 4–6 month, and 7–12 month time points across the time periods.

After hospital discharge, the patients were followed up for a median of 1.85 years (interquartile range, 0.44 to 5.08 years). Pneumococcal vaccines were administered to 16.6% (557/3356) of the eligible patients and 11.7% (121/1031) of the ineligible patients. Among the eligible patients, 12.1% (405/3356) received one dose, and 4.5% (152/3356) received two doses. The median time to post-discharge pneumococcal vaccination was 10.6 months (interquartile range, 2.4 to 29.3 months) for the first dose and 28.3 months (interquartile range, 18.8 to 45.5 months) for the second dose. The percentage of eligible patients receiving pneumococcal vaccines shortly after hospital discharge was low, ranging from 2.4% to 5.7% in the baseline period to 0.5% to 2.1% in the COVID-19 period and 0.8% to 4.5% in the post-COVID-19 period for time points within the initial 12 months (Figure 3). Notably, the proportions vaccinated within 3 months, within 4 to 6 months, and within 7 to 12 months post-discharge were significantly lower in the patients hospitalized during the COVID-19 pandemic compared to the baseline and post-COVID-19 periods (*p* < 0.001 for all comparisons across the time periods at the three post-discharge time points). A rebound was observed in the post-COVID-19 period, but the vaccination rates were still numerically lower than those in the baseline period (Figure 3). Vaccination rates beyond 12 months post-discharge were not analyzed across the three periods because of the uneven duration of follow-up.

Among the eligible patients, the overall pneumococcal vaccination uptake rate increased from 20.5% at admission to 26.7% post-discharge, with partial vaccination increasing from 12.5% to 16.9% and full vaccination increasing from 8.1% to 9.8% (Figure S1). The post-discharge vaccination uptake rates varied depending on the patients’ admission year in the study period (Figure S1). The partial and full vaccination rates ranged from 11.9% to 20.8% and 11.6% to 18.8%, respectively, with 64.7% to 75.6% of the patients remaining unvaccinated. The full vaccination uptake rate among those aged 18–64 with risk conditions, those aged ≥65 without any risk conditions, and those aged ≥65 with risk conditions was 7.3%, 33.9%, and 12.4%, respectively (Figure 2).

Data on the type of pneumococcal vaccine were available for 98.9% (1452/1468) of the patients who received at least one dose, with 946 patients receiving one dose and 506 patients receiving two doses. Among those who had one dose, 54.5% (516/946) had 23vPPV, 43.9% (415/946) had PCV13, and 1.6% (15/946) had PCV15. For patients who had two doses, 54.5% (276/506) had 23vPPV followed by PCV13, and 45.5% (230/506) had PCV13 followed by 23vPPV.

### 3.2. Missed Opportunities for Pneumococcal Vaccination

Overall, 93.8% (5174/5517) of the patients hospitalized for pneumococcal infection had previously received medical care in public hospitals. Among them, 82.7% (4568/5517) had been hospitalized at least once, and 92.5% (5105/5517) had visited outpatient clinics (Table 1). The rate of prior hospitalization was higher in the patients aged ≥65 years (77.8–95.3%) compared to those aged 18–64 y (59.4% to 82.2%). In the subgroups, the median number of prior hospitalizations ranged from 3 to 10. The patients aged ≥65 years with risk conditions had the highest number of previous hospitalizations (median 10, IQR 5–18), while those aged 18–64 without risk conditions had the lowest (median 3, IQR 1–6). Across the subgroups, the median number of previous outpatient visits ranged from 26 to 108. The patients aged ≥65 years with risk conditions had the highest number of previous outpatient visits (median 108), while those aged 18–64 years without risk conditions had the lowest (median 26) (Table 1).

Among the surviving patients, 98.0% (4298/4387) had sought medical care in public hospitals following their pneumococcal hospitalization. Of these patients, 84.7% (3716/4387) had experienced at least one subsequent hospitalization, and 93.4% (4097/4387) had attended outpatient clinics (Table 1). Within the subgroups, the median numbers of subsequent hospitalizations ranged from 3 to 5, while outpatient visits ranged from 19 to 34.

### 3.3. Factors Associated with Pneumococcal Vaccination

**Figure 4:**
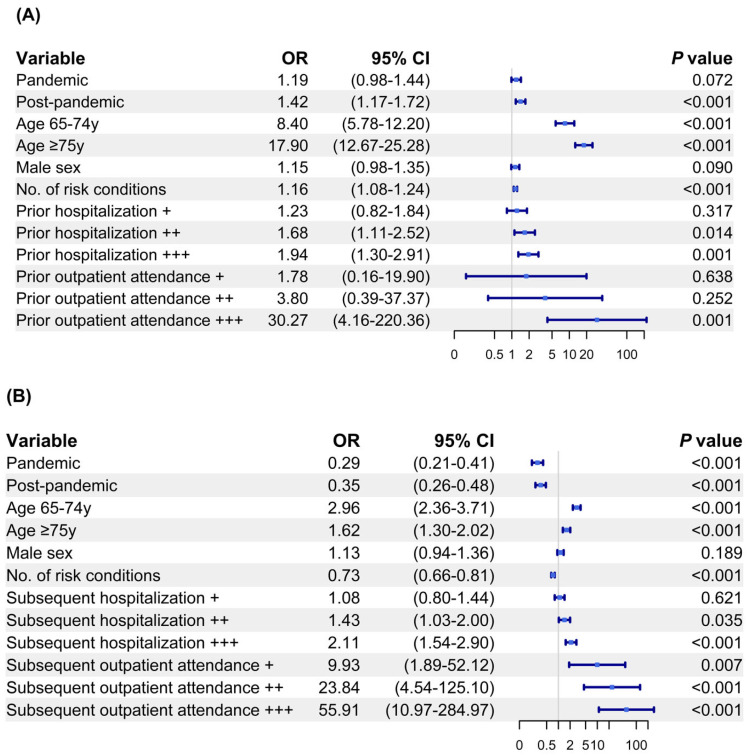
Multivariate logistic analysis of factors associated with pneumococcal vaccination (**A**) at the time of hospitalization for pneumococcal infection and (**B**) for patients surviving the pneumococcal infection hospitalization. Prior hospitalization and outpatient attendance are graded as + (1 to 5 times), ++ (6 to 10 times), and +++ (more than 10 times).

Prior to hospitalization, a higher uptake of pneumococcal vaccination was observed in the post-pandemic period (OR 1.42), among patients aged 65–74 (OR 8.40) and ≥75 years (OR 17.90), among those with a higher number of risk conditions (OR 1.16), among those previously hospitalized 5–10 times (OR 1.68) and ≥10 times (OR 1.94), and among those with previous clinic attendance of ≥10 times (OR 30.27) (Figure 4A).

Conversely, the pandemic period (OR 0.29), the post-pandemic period (OR 0.35), and an increased number of risk conditions (OR 0.73) were significantly associated with lower pneumococcal vaccination uptake post-hospitalization (Figure 4B). On the other hand, a higher pneumococcal vaccination uptake was associated with older ages (65–74 years, OR 2.96; ≥75 years, OR 1.62), subsequent hospitalization for 5–10 times (OR 1.43) and ≥10 times (OR 2.11), and subsequent clinic attendance (1–5 times, OR 9.93; 5–10 times, OR 23.84; ≥10 times, OR 55.91).

## 4. Discussion

This study revealed low uptake of the pneumococcal vaccine and highlighted frequent missed opportunities for pneumococcal vaccination among adults hospitalized for pneumococcal disease. Among patients eligible for vaccination, only one in five received at least one dose, but less than half of this group were considered to be appropriately vaccinated (i.e., fully vaccinated). The primary reason for this discrepancy is that patients with a risk-based indication require two doses (PCV 13 + 23vPPV or PCV15 + 23vPPV), yet many had only received a single dose of either PCV13 or 23vPPV. The vaccination uptake in the patient population in this study is notably lower than that of the general elderly population, which was 34% in 2016 and 42% in 2023 [15]. The majority of patients who developed pneumococcal disease had multiple missed vaccination opportunities in both outpatient and inpatient settings. In Hong Kong, a qualitative study on the pneumococcal vaccination strategy highlighted that healthcare providers may face challenges due to time and logistical constraints, as well as ill-defined criteria for risk-based patient eligibility [18]. Furthermore, the patient barriers included concerns about vaccine efficacy, poor understanding of the disease, and vaccine schemes [18].

To our knowledge, this is the first study to explore missed opportunities of pneumococcal vaccinations post-recovery from pneumococcal disease. In the study cohort, the uptake of pneumococcal vaccines post-discharge remained low, with eligible patients showing an increase to 32.2% for receiving at least one dose and 16.5% for achieving full immunization. Similarly, multiple vaccination opportunities were missed in both the outpatient and inpatient settings for many patients. The timing of post-discharge vaccination was also found to be suboptimal. Among those who received the pneumococcal vaccine after recovering from an infection, the proportion of vaccines administered shortly after discharge (within 3 months) was low. This could be attributed to the low perceived benefit of the pneumococcal vaccine and the lack of recommendation during hospitalization [37,38]. Currently, there are no specific timing recommendations for post-infection pneumococcal vaccination [9,13]; however, it is generally understood that vaccination can occur once recovery from the disease is achieved. We posit that the vaccination rate in this patient population can be enhanced by incorporating pneumococcal immunization into the discharge plan. Implementing computer-based physician standing orders upon discharge can be valuable, as supported by findings from a randomized study [39].

Our data showed that the administration of pneumococcal vaccination to patients post-discharge decreased during the COVID-19 pandemic. Despite a rebound in the initial two years post-COVID-19, the vaccination rates were still lower than those in the baseline period. In a telephone survey of community-living adults aged ≥65 years in Hong Kong, the self-reported uptake rate of pneumococcal vaccine was found to increase during and after the COVID-19 pandemic, indicating that there was a change in health-seeking behavior. Disruption of healthcare services, especially the overloading of public hospital services during COVID-19, may explain these findings [31,40].

In our cohort, 19% of the patients hospitalized for pneumococcal infection were deemed ineligible for the pneumococcal vaccine, with half of this group aged 50 to 64 years. Pneumococcal pneumonia has been estimated to account for 13% of all hospitalized pneumonia cases [13]. In Hong Kong, the annual burden of all pneumonia hospitalizations has been estimated to be approximately 950 per 100,000 population, which is comparable to those reported in the United States [41,42]. In our locality, among all pneumonia hospitalizations, the proportion of patients aged 50–64 years (10%) was similar to those aged 65–74 years (12%) and was higher than those in other age groups, but only lower than those aged ≥75 years [42]. In October 2024, the Advisory Committee on Immunization Practices (ACIP) in the United States revised the age-based recommendation for pneumococcal vaccination in adults from ≥65 years to ≥50 years [13]. A single dose of PCV, instead of 23vPPV, is recommended because of improved immunogenicity and the induction of the memory response [3,7]. If PCV15 is administered, a single dose of 23vPPV should be administered at least one year after the PCV15 dose. As our data illustrate, the need for two doses could impose a logistical burden on the patient and provider, leading to large proportions of patients partially immunized. Additionally, administration of the two types of vaccine could be suboptimal, with 23vPPV followed by PCV.

PCV20 included seven additional serotypes (8, 10A, 11A, 12F, 15B, 22F, 33F) that are not contained in PCV13 [13]. In Hong Kong, from 2015 to 2024, a total of 65 adult cases of IPD were reported to be caused by these seven additional serotypes [25]. Among these cases, only a small proportion (26%) of the cases were caused by the two additional serotypes (22F and 33F) included in PCV15 [25]. A recent systematic review of the cost-effectiveness of adult pneumococcal vaccination strategies concluded that PCV20 used alone was cost-saving or cost-effective compared to 23vPPV alone, PCV15 + 23vPPV, or no vaccination [43]. Taking these factors into consideration, replacing the current recommendation (one dose 23vPPV or PCV15 + 23vPPV) with a single dose of PCV20 and lowering the age-based indication to ≥50 years could simplify the logistics, provide broader coverage, and prevent more cases of pneumococcal disease [44].

The low pneumococcal vaccination uptake and frequent missed opportunities highlighted in the current study carry significant public health implications. The existing strategies intended to promote the uptake of the pneumococcal vaccine in the at-risk population should be reviewed. In many countries and regions, governments have introduced personalized electronic health accounts that integrate a multitude of hospital and clinic processes. These systems empower patients and healthcare providers to make informed decisions and address patients’ health needs effectively. Leveraging electronic health information systems can facilitate timely, convenient, and personalized pneumococcal vaccination for individuals.

Some limitations of this study should be acknowledged. Firstly, this study did not account for vaccines administered in the private sector, potentially leading to an underreporting of the actual vaccine uptake. In a telephone survey of older adults, the pneumococcal vaccination uptake rates for at least one dose before (17.3%), during (28.3%), and after (35.5%) the COVID-19 pandemic were low and comparable to those reported in this study [37]. Secondly, the vaccination period prior to hospitalization was assessed up to January 2005. The period prior was not included or evaluated because of the limited records available from the CDARS system, with the possibility that patients who received vaccination in this period were included as unvaccinated.

## 5. Conclusions

This study, conducted on a territory-wide cohort of patients hospitalized for pneumococcal disease, revealed low uptake of the pneumococcal vaccine before hospitalization for the disease, which continued to be suboptimal post-discharge. Uptake of the vaccine post-discharge further decreased during and post-COVID-19 pandemic. Numerous vaccination opportunities were missed in both the inpatient and outpatient settings. These findings are crucial in guiding the selection of pneumococcal conjugate vaccine formulations and improving vaccination strategies.

## Figures and Tables

**Table 1:** Hospital care and outpatient attendance in patients with pneumococcal infection.

Age Group	Outcome	*n*	Previous Hospitalization		Previous Outpatient Attendance		Subsequent Hospitalization		Subsequent Outpatient Attendance	
			Total (%)	No	Total (%)	No	Total (%)	No	Total (%)	No
18–64 y old without risk conditions	Survived	983	576 (58.6)	3 (1–6)	794 (80.8)	35 (12–81)	659 (67.0)	3 (1–7)	894 (90.9)	19 (5–47)
	Died	65	47 (72.3)	7 (3–17)	56 (86.2)	25 (7–66)	-	-	-	-
	Subtotal	1048	623 (59.4)	3 (1–6)	850 (81.1)	26 (7–70)	-	-	-	-
18–64 y old with risk conditions	Survived	539	444 (82.4)	8 (3–17)	489 (90.7)	71 (30–142)	453 (84.0)	5 (2–13)	517 (95.9)	34 (11–73)
	Died	134	109 (81.3)	6 (3–13)	112 (83.6)	69 (21–110)	-	-	-	-
	Subtotal	673	553 (82.2)	8 (3–16)	601 (89.3)	70 (28–134)	-	-	-	-
≥65 y old without risk conditions	Survived	1027	799 (77.8)	4 (2–9)	948 (92.3)	68 (28–118)	890 (86.7)	4 (2–9)	966 (94.1)	32 (19–61)
	Died	256	199 (77.7)	5 (2–9)	231 (90.2)	65 (29–117)	-	-	-	-
	Subtotal	1283	998 (77.8)	4 (2–9)	1179 (91.9)	67 (28–118)	-	-	-	-
≥65 y old with risk conditions	Survived	1838	1749 (95.2)	10 (5–18)	1809 (98.4)	108 (67–165)	1714 (93.3)	5 (2–10)	1720 (93.6)	23 (9–52)
	Died	675	645 (95.6)	9 (5–17)	666 (98.7)	109 (60–168)	-	-	-	-
	Subtotal	2513	2394 (95.3)	10 (5–18)	2475 (98.5)	108 (65–166)	-	-	-	-
All patients	Survived	4387	3568 (81.3)	7 (3–15)	4040 (92.1)	79 (32–138)	3716 (84.7)	4 (2–10)	4097 (93.4)	25 (9–56)
	Died	1130	1000 (88.5)	8 (4–15)	1065 (94.2)	96 (44–152)	-	-	-	-
	Total	5517	4568 (82.8)	7 (3–15)	5105 (92.5)	82 (33–141)	-	-	-	-

## Data Availability

The data are unavailable due to privacy or ethical restrictions.

## Supplementary Materials

The following supporting information can be downloaded at https://www.mdpi.com/article/10.3390/vaccines13050541/s1: Figure S1: Uptake of pneumococcal vaccination among eligible patients in the study cohort (A) at the time of hospitalization for pneumococcal infection (before) and (B) at the censor date after discharge for pneumococcal infection (after).

## Abbreviations

The following abbreviations are used in this manuscript:

IPD	invasive pneumococcal disease
23vPPV	23-valent polysaccharide pneumococcal vaccine
PCV	pneumococcal conjugate vaccine
COVID-19	Coronavirus disease
CDARS	Clinical Data Analysis and Reporting System
ORs	odd ratios
