# Supplementary material for: Pneumococcal Vaccine Uptake in Adults Before and After Hospitalization for Pneumococcal Infections in Hong Kong, 2015 to 2024
Source: Vaccines (Basel). 2025 May 19;13(5):541. doi: 10.3390/vaccines13050541 (PMC12115717; doi:10.3390/vaccines13050541)

Supplementary file

Pneumococcal Vaccine Uptake in Adults Before and After Hospitalization for Pneumococcal Infections in Hong Kong, 2015 to 2024

King-Pui Florence Chan [1], Ting-Fung Ma [2], James Chung-Man Ho [1], Ivan Fan-Ngai Hung [1], Mary Sau-Man Ip [1] and Pak-Leung Ho [3,4,*]

Figure S1. Uptake of pneumococcal vaccination among eligible patients in the study cohort (A) at the time of hospitalization for pneumococcal infection (before), and (B) at the censor date after discharge for pneumococcal infection (after).

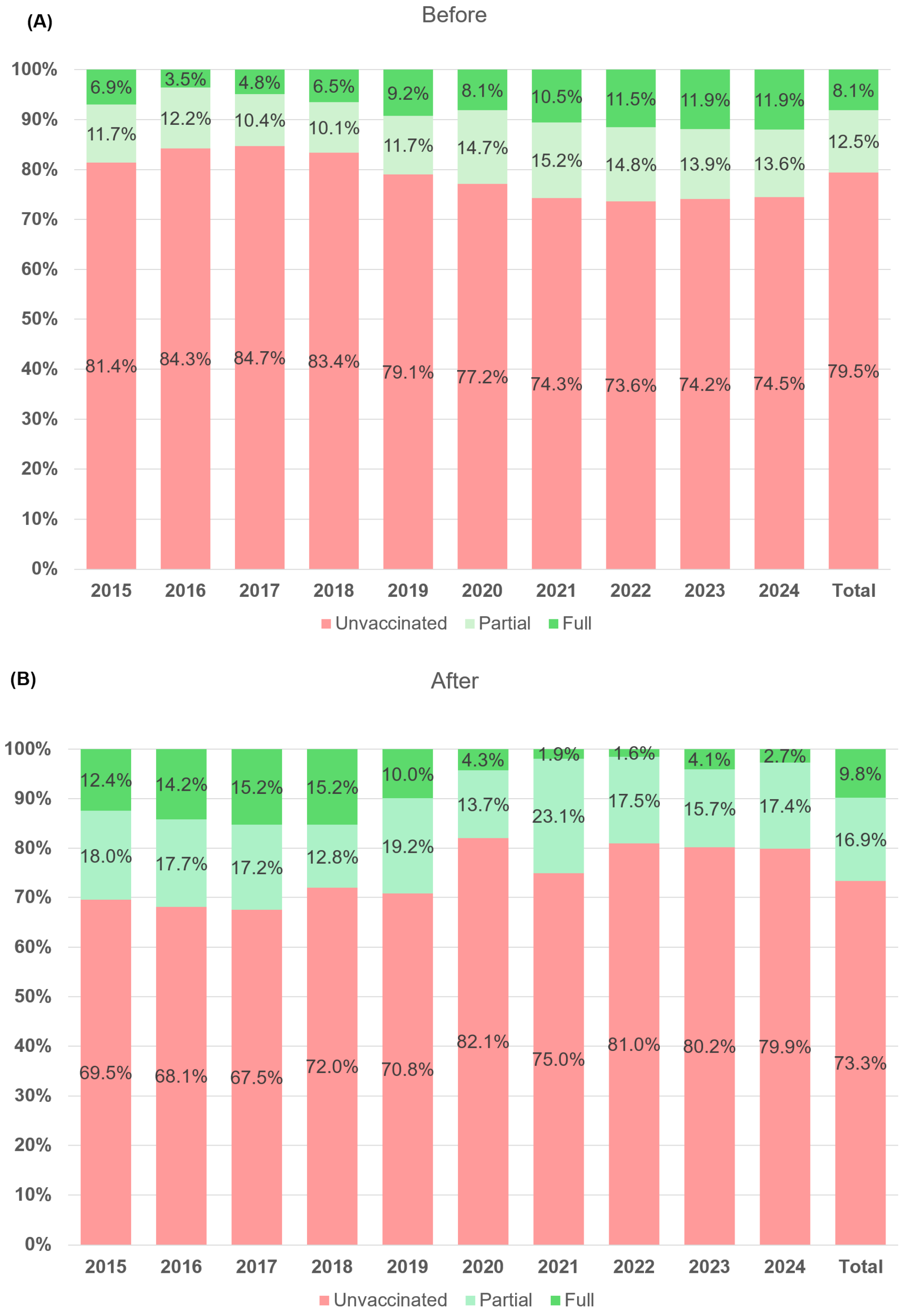

Supplement: Supplementary file 1 [file vaccines-13-00541-s001.zip › vaccines-3632469-supplementary.pdf]
